# Recent Advancements in the Clinical Pathway of Respiration-Synchronized Hypoglossal Nerve Stimulation Therapy for Obstructive Sleep Apnea

**DOI:** 10.3390/jcm14228241

**Published:** 2025-11-20

**Authors:** Dorine Van Loo, Eldar Tukanov, Marijke Dieltjens, Sara Op de Beeck, Eli Van de Perck, Johan Verbraecken, Olivier M. Vanderveken

**Affiliations:** 1 Translational Neurosciences, Faculty of Medicine and Health Sciences, University of Antwerp, 2610 Wilrijk, Belgium; 2 Department of ENT, Head and Neck Surgery, Antwerp University Hospital, 2650 Edegem, Belgium; 3 Multidisciplinary Sleep Disorders Centre, Antwerp University Hospital, 2650 Edegem, Belgium; 4 Research Group LEMP, Faculty of Medicine and Health Sciences, University of Antwerp, 2610 Wilrijk, Belgium

**Keywords:** hypoglossal nerve stimulation, upper airway stimulation, HGNS, UAS, obstructive sleep apnea, post-implant care management

## Abstract

**Background**: Respiration-synchronized hypoglossal nerve stimulation (HGNS) is an effective treatment option for selected patients with obstructive sleep apnea (OSA). While literature on the standardization of the post-implant care pathway for patients treated with HGNS therapy remains limited, the growing global use of HGNS has made structured post-implant care management increasingly important. **Methods**: This narrative review summarizes the advancements related to the clinical pathway for respiration-synchronized HGNS therapy over the past 5 years, with a special focus on post-implant care management. **Results**: Selection criteria for respiration-synchronized HGNS are changing as new clinical findings emerge, including both anatomical and non-anatomical markers. Evidence suggests that adopting single-amplitude, full-night sleep testing may provide a more reliable assessment of HGNS treatment efficacy. Several studies have described optimization strategies for patients with suboptimal HGNS therapy response or therapy-related discomfort, including the use of awake endoscopy and drug-induced sleep endoscopy for advanced HGNS programming and the use of combination therapy, although data remains limited. **Conclusions**: The clinical care pathway for respiration-synchronized HGNS continues to evolve, including patient selection, evaluation of treatment success, and strategies for managing incomplete responders or patients with therapy-related discomfort. Nonetheless, addressing insufficient treatment responses remains a significant challenge and a key area for future research and clinical optimization.

## 1. Introduction

With over 100,000 patients implanted worldwide, respiration-synchronized hypoglossal nerve stimulation (HGNS) has become a clinically effective treatment option for selected patients with obstructive sleep apnea (OSA) who are unable to tolerate continuous positive airway pressure (CPAP) or mandibular advancement device therapy (MAD), or for whom these therapies provide insufficient benefit. Over the past 30 years, extensive research has been conducted leading to continuous advancements in the HGNS system and its applications. The existing body of literature addressing the safety and efficacy of HGNS therapy is substantial, including long-term studies that clearly demonstrate the efficacy and safety of the system in selected patients with moderate to severe OSA [1,2,3,4,5]. However, there is limited literature fully standardizing the post-implant care pathway for patients treated with HGNS therapy [6,7].

With the growing number of patients treated with HGNS therapy globally, post-implant care management is playing an increasingly pivotal role. Data from the ADHERE registry, a multi-center observational study collecting data on up to 5000 OSA patients treated with HGNS therapy in routine clinical practice, showed that 69% of patients met the Sher20 criteria (≥50% reduction in baseline AHI and a postoperative AHI < 20 events/h) at 1-year post-implantation, leaving a substantial subgroup of patients with a partial or incomplete response [8]. Additionally, about 19% of patients reported therapy-related discomfort (defined as stimulation-related discomfort, insomnia/arousal or tongue abrasion). In this subgroup of the cohort, therapy adherence was lower, and the mean AHI at 1-year post-implantation was significantly higher than in patients with no reported discomfort [5]. For these patient groups, a standardized clinical pathway and close monitoring are essential.

**Figure 1:**
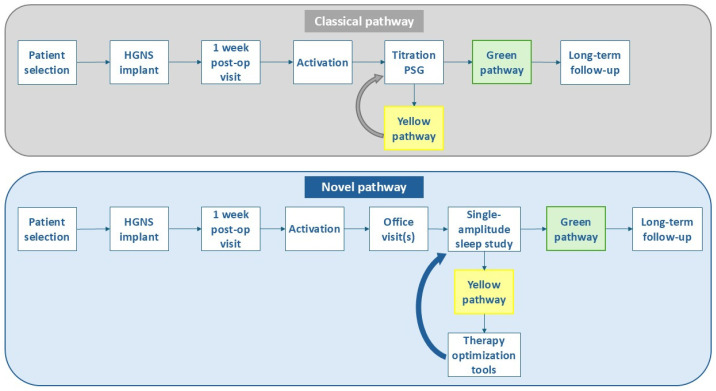
Overview of the classical versus novel clinical pathway for respiration-synchronized HGNS. Device activation occurs approximately 4–6 weeks post-implantation, followed by an adaptation period of at least 3 months. The novel pathway includes multiple office visits to check on the adherence, comfort and subjective improvement. There has been a shift away from standard titration PSGs (classical pathway) toward single-amplitude, full-night efficacy sleep studies (novel pathway). Patients with adequate therapy usage that meet Sher15 criteria (≥50% AHI reduction and on-therapy AHI <15/h) follow the green pathway with long-term follow-up. Patients with an incomplete response and/or therapy-related discomfort follow the yellow pathway. While the classical pathway mostly relied on advanced titration PSGs, the novel pathway incorporates different therapy optimization tools (awake endoscopy, drug-induced sleep endoscopy and combination therapy) for incomplete HGNS responders or patients with therapy-related discomfort. After therapy has been optimized, a follow-up single-amplitude sleep study can be conducted to assess treatment efficacy. Abbreviations: HGNS = hypoglossal nerve stimulation; PSG = polysomnography.

In recent years, the selection criteria for HGNS have undergone refinement to better identify suitable candidates. At the same time, post-implant care has advanced, including a shift away from a standard titration polysomnography (PSG) toward single-amplitude, full-night efficacy sleep studies and the implementation of different optimization tools for patients with suboptimal treatment response or therapy-related discomfort (Figure 1).

This narrative review aims to update and extend previous literature by summarizing the advancements related to the clinical pathway, including patient selection, implantation, activation, titration and long-term follow-up, for respiration-synchronized HGNS therapy over the past 5 years, with a special focus on post-implant care management.

## 2. Methods

Relevant literature was identified through a targeted search of PubMed up to 31 August 2025, focusing on articles published in the last five years, using key words including “hypoglossal nerve stimulation”, “upper airway stimulation”, “clinical pathway”, “post-implant care”. Articles were selected based on their relevance to advancements in the clinical pathway of respiration-synchronized HGNS, with only articles including adult patients (≥18 years) considered.

## 3. Patient Selection

In recent years, the association between various baseline characteristics and HGNS treatment response has been investigated [9]. However, the results show considerable variation, highlighting the need for further research to explore the predictive value of baseline characteristics for HGNS outcomes. At present, the only airway collapse pattern formally contraindicated for HGNS is complete concentric collapse (CCC) of the soft palate [10]. Complete oropharyngeal or lateral wall collapse has gained increasing interest, as multiple studies have shown that its presence also predicts lower response to HGNS [11,12,13]. In addition to anatomical markers, pathophysiological traits also play an important role in HGNS outcomes. It has been demonstrated that a high arousal threshold, low loop gain, and high muscle responsiveness at baseline are associated with a successful response to HGNS therapy [14]. Regarding the impact of insomnia on HGNS treatment outcomes, there is conflicting data on adherence and therapy success [15,16,17,18]. A cross-sectional study showed that the presence of anxiety, depression and/or emotional distress may contribute to reduced HGNS adherence [19].

The selection process for HGNS therapy includes comprehensive screening to determine whether a patient meets the eligibility criteria. The following criteria were approved by the FDA in 2014: an apnea-hypopnea index (AHI) ranging from 20 to 65 events/h diagnosed by a recent polysomnography (PSG), a body-mass index (BMI) of less than 32 kg/m^2^, a combined central and mixed AHI of less than 25%, CPAP intolerance, and the absence of CCC at the soft palate level, as determined during drug-induced sleep endoscopy (DISE) [1,20].

Previously, the lower AHI limit had been reduced from 20 events/h to 15 events/h [21]. Recent findings have led to FDA approval for an increase in the upper limit of the AHI from 65 events/h to 100 events/h, as well as a raise in the BMI limit from 32 kg/m^2^ to 40 kg/m^2^ [5,22]. However, it should be noted that patients with BMI ≥ 32 kg/m^2^ have lower odds of achieving therapeutic success [23,24,25]. Furthermore, FDA approval was also granted for patients aged between 18 and 21 years, and CPAP-intolerant pediatric Down syndrome patients aged 13–18 years with the following criteria: AHI between 10 and 50 events/hour, absence of CCC at the level of the soft palate, and not eligible for adenotonsillectomy or other therapies [26,27]. However, the criteria for reimbursement in Europe differ from one country to another.

## 4. Implantation

**Figure 2:**
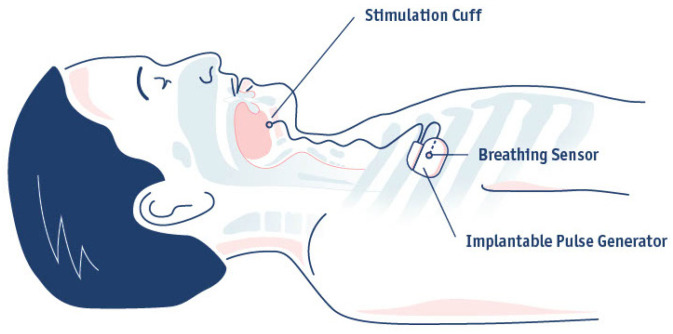
Overview of the respiration-synchronized HGNS system of Inspire Medical Systems (Inspire IV). The respiration-synchronized HGNS system (Inspire IV) consists of three implantable components: the stimulation lead with cuff electrode, the implantable pulse generator (IPG), and the breathing sensor.

The respiration-synchronized HGNS system of Inspire Medical Systems (Inspire IV) consists of three implantable components: the stimulation lead with cuff electrode, the implantable pulse generator (IPG), and the breathing sensor (Figure 2). Nowadays, the surgical technique has been refined to two incisions instead of three [28]. The breathing sensor is placed between the internal and external intercostal muscles in the second intercostal space instead of the fifth, by using the same incision as for the placement of the IPG. A selective nerve integrity monitoring (NIM) system is used for the placement of the cuff electrode around the protruding branches of the hypoglossal nerve, including all extrinsic protrusors (oblique and horizontal genioglossi), all intrinsic stiffeners (transversal and vertical) and the first cervical nerve (C1) which innervates the geniohyoid muscle [29]. The IPG synchronizes the stimulation with breathing and contains the battery, which needs to be replaced approximately 8–12 years post-implantation. A detailed overview of the implantation has already been described in literature [6,29]. A post-operative follow-up is scheduled approximately 7–10 days post-implantation, during which the wound healing is evaluated.

There have been several advancements in the respiration-synchronized HGNS system. In the current CE-marked version of the system, the implanted leads use silicone insulation, enhancing durability and longevity [30]. Additionally, the current sensing lead is shorter, as it is now implanted through the same incision used for the IPG. Furthermore, additional magnetic resonance imaging (MRI) scan conditions have been approved for all patients with the Inspire IV system, which is full-body MRI conditional at 1.5T [30,31]. Recently, the FDA approved the fifth-generation of the Inspire system which includes a smaller IPG and respiratory sensor integrated into the IPG thereby alleviating the need for a separate sensing lead [32].

## 5. Activation, Education and Acclimation

Approximately 4 to 6 weeks post-implantation, the HGNS device is activated during a daytime clinical visit. Using the physician programmer, which consists of a tablet and a telemetry unit, wireless communication with the IPG is established through short-range radiofrequency telemetry. Then, the sensation threshold (the minimum stimulation voltage that the patient can feel) and the functional threshold (the voltage that results in tongue protrusion that exceeds the border of the lower incisors) are determined [6]. Furthermore, the waveform is assessed to confirm proper breathing sensor function. In general, the device is set at the functional threshold in standard settings (electrode configuration [+−+], pulse width 90 μs, frequency 33 Hz), if comfortable for the patient [6]. During this visit, the patient is instructed in the use of the patient remote, which allows them to switch the therapy on and off, to pause the therapy, and to make small adjustments to the stimulation strength within a pre-defined range. A stepwise self-titration protocol is instructed, in which the patient has to gradually increase the stimulation strength at home by one step per week, and to stop increasing if sleep quality and snoring is improved or if any discomfort occurs. “All night, every night” usage is encouraged as this is essential for acclimation to the therapy [6,7].

The adaptation phase involves acclimating to the therapy and gradually self-titrating the amplitude to optimize symptom improvement and comfort. Typically, the adaptation phase lasts approximately three months, but this can vary significantly between patients. A potential pitfall of self-titration is over-titration of the amplitude, leading to worsening of OSA symptoms [33]. Therefore, close clinical monitoring in the initial stages of therapy usage is essential as this will significantly impact long-term outcomes. The first follow-up is recommended within one month after activation, either as a phone call or in-office visit, to check on the adherence, comfort and subjective improvement [7].

Therapy-related discomfort should be addressed early to improve HGNS adherence and outcomes. A recent retrospective study showed that patients requiring post-activation adjustment due to therapy discomfort have significantly poorer long-term outcomes [34]. Personalization of therapy can be achieved by adjusting parameters, such as start delay and pause time, based on individual patient needs. Additionally, modifying pulse width and rate can help to optimize comfort. Standard HGNS settings typically use a pulse width of 90 μs and a frequency of 33 Hz. However, by increasing the pulse width to 120 μs and the frequency to 40 Hz, the amplitude can be reduced while maintaining a similar tongue protrusion, enhancing patient comfort [35]. Alternative combinations, such as 120 μs/33 Hz or 90 μs/40 Hz can also be explored and fine-tuned based on patient feedback.

A retrospective cluster analysis of therapy usage in the first 90 days after device activation in more than 2000 patients identified six distinct groups based on the usage time, therapy pauses, hours from midnight the therapy was turned ON and OFF, and percentage of missing days [36]. These clusters range from excellent, consistent use to good use with occasional missed days, delayed start, and early termination, and finally to variable use characterized by recurring missed days or frequent pauses. This study showed that most of the patients (85%) are excellent or good users with favorable nightly usage (>6 h per night) [36]. The findings of this study indicate that understanding the distinct patterns of adherence and the factors driving them is more important than simply measuring usage time. Furthermore, personalized interventions such as therapy reprogramming and/or management of comorbid sleep conditions may be implemented based on the usage pattern of the patient [36]. Therefore, the use of a cloud-based monitoring system is of great value as it provides information on the time the device was turned on, turned off, paused, and on the therapy amplitude used. This information is remotely accessible to the physician and directly available to the patient through a smartphone application. A prospective cohort study by Yu et al. compared therapy adherence between patients using the new Bluetooth-connected remote (n = 126) and the original remote (n = 121). The study showed that the use of a connected sleep remote was associated with higher adherence in terms of nights with therapy usage at 90 days, suggesting that the smartphone application creates positive reinforcement [37].

## 6. Defining HGNS Treatment Success

Three key parameters should be considered when evaluating HGNS therapy success: therapy usage, symptom response and sleep study metrics. If the patient reports comfortable therapy usage along with symptomatic improvement, a follow-up sleep study is typically scheduled three months post-activation [7]. Historically, the standard of care involved a type I titration PSG, which assessed the device settings and, if necessary, allowed for adjustment of the stimulation strength based on observed respiratory events and/or snoring [6]. However, overnight titration PSG has some disadvantages. Considering the growing patient population, the performance of these overnight titrations can become logistically challenging and labor-intensive. Recently, the feasibility of using a daytime PSG as an alternative to a conventional overnight PSG for titration of HGNS therapy was demonstrated [38].

The outcome of this titration night is generally reported by using treatment AHI, which is defined as the AHI under optimal therapeutic settings and is commonly used across numerous HGNS publications to assess therapy efficacy. Nevertheless, there are no standardized guidelines describing minimum sleep duration, body position or sleep stage required to calculate this treatment AHI [39]. A recent retrospective study including 61 patients has shown that the postoperative titration AHI is significantly lower than the AHI determined by using a single amplitude, full-night home sleep test (HST), and thus results in a significant overestimation of treatment effect [40]. Furthermore, it is important to consider whether the patient can tolerate the optimal settings found during the titration study.

Because of the limitations mentioned above, there has been a shift away from standard titration PSGs toward single-amplitude (one fixed stimulation level), full-night efficacy sleep studies, being either a type I PSG or HST. This approach is also referred to as “patient-directed therapy titration” based on comfort and symptom improvement [40]. The aforementioned strategy is built on the concept of the three-dimensional mean disease alleviation, which integrates the subjective patient response alongside the overnight adherence to the therapy and objective treatment efficacy [41].

Two studies have compared the use of in-laboratory titration PSG versus HST to assess HGNS efficacy. Steffen et al. showed no significant differences between PSG- or HST-controlled post-implantation therapeutic effects at 2 months and long-term follow-ups [42]. In a prospective, multicenter study conducted by Kent et al., 60 patients were randomized to undergo either a standard in-laboratory titration PSG or an efficacy HST at 3 months post-activation. At 6 months post-activation, both groups demonstrated similar improvements in objective and subjective OSA outcomes, suggesting that a titration PSG may not be required [43]. However, the use of HSTs also has some limitations. It should be considered that HSTs can underestimate the residual disease burden as it calculates the AHI based on recording time and not sleep time. Additionally, it is uncertain whether HGNS therapy was active during the whole sleep time. Kaffenberger et al. have proposed a nomenclature that specifies the type of therapeutic sleep study that was used to determine treatment success [44].

In addition to objective outcomes determined during a sleep study, symptom-related outcomes should also be evaluated by using dedicated questionnaires such as the Epworth Sleepiness Scale (ESS) and Functional Outcomes of Sleep Questionnaires (FOSQ). Implementing these patient-reported outcome measures (PROMs) is essential for advancing patient-centered care [45].

Another important aspect of treatment evaluation is the effect of OSA therapy on reducing OSA-related comorbidities. Literature on the cardiovascular outcomes of HGNS therapy remains limited. A retrospective sub-analysis on HGNS responders from the STAR trial showed that HGNS appears to reduce heart rate variability during sleep and that this reduction was not affected by a 1-week withdrawal period [46]. Walia et al. investigated the comparative changes in blood pressure between HGNS and CPAP in patients with moderate to severe OSA, showing that HGNS-treated patients did not have significant changes in systolic blood pressure, diastolic blood pressure or mean arterial pressure at 4 months follow-up, in contrast to CPAP therapy [47]. In addition, a sham-controlled HGNS randomized clinical trial involving 60 patients showed no significant differences in mean 24 h systolic blood pressure and other cardiovascular measures between sham and active HGNS therapy [48]. Future trials are needed to examine both the short- and long-term cardiovascular outcomes of HGNS.

Patients will be directed to either a green or yellow pathway, depending on their therapy usage, sleep study outcomes and symptom improvement. Patients with adequate therapy usage of more than 4 h per night that meet Sher15 criteria (≥ 50% reduction in AHI and an on-therapy AHI of <15 events/h) will follow the green pathway with long-term follow-up. Failure to meet one of these criteria or both will direct patients to the yellow pathway. As described by Soose et al., several yellow pathway subtypes exist [7]. Patients may have good outcome measures but suboptimal adherence, or good adherence with suboptimal symptom and/or AHI reduction, or a combination of both. However, it is important to address therapy adherence early and before scheduling the patient for a follow-up sleep study. Patients directed onto the yellow pathway necessitate further therapy optimization, for which different tools are currently available.

## 7. Optimization Tools for HGNS Patients in the Yellow Pathway

**Figure 3:**
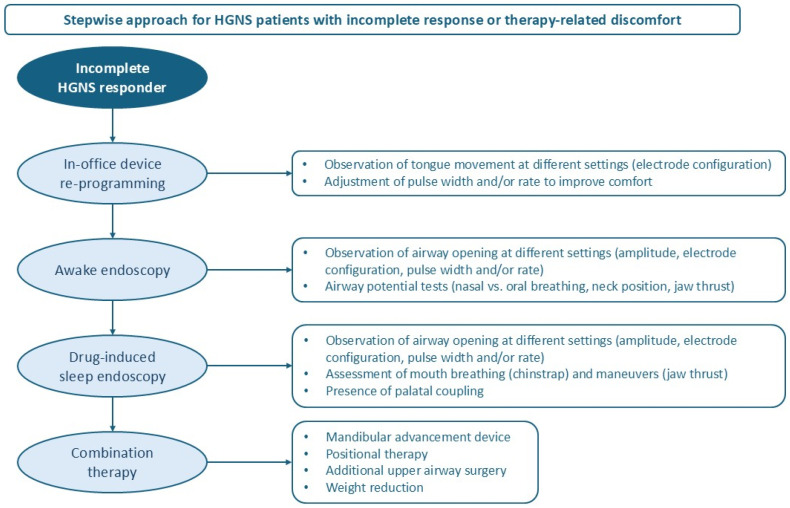
Clinical pathway for HGNS patients in the yellow pathway with incomplete response or therapy-related discomfort. Different optimization tools are currently available which can be used in a stepwise approach to optimize patients with an incomplete response or therapy-related discomfort. Abbreviations: HGNS = hypoglossal nerve stimulation.

HGNS patients in the yellow pathway follow a stepwise approach to optimize treatment (Figure 3). A summary of the included studies in this section can be found in Table 1.

### 7.1. In-Office Device Reprogramming

The first step to be taken for HGNS patients in the yellow pathway is in-office device reprogramming based on observation of tongue movement. In an early retrospective study by Heiser et al., patients with bilateral tongue protrusion demonstrated a greater AHI reduction than patients with mixed activation [49]. Steffen et al. observed changes in tongue motion patterns with different electrode configuration in 58.8% of patients, of which the alternation between right and bilateral protrusion was observed most frequently (73.5%) [50]. A prospective study by Pawlak et al. demonstrated that both the amplitude and electrode configuration influence airway patency, as observed during awake endoscopy and DISE [51].

The majority of the device changes are confined to the stimulation parameters. Respiratory sensing settings are rarely adjusted in routine clinical practice. Kent et al. have proposed an algorithm for office-based respiratory sensing re-programming to optimize HGNS respiratory entrainment. In a case series, improvements in patient comfort and HGNS efficacy were shown with office-based changes to respiratory sensing parameters that affect stimulation timing. This suggests that there might be a small group of non-responders that could benefit from adjusting respiratory entrainment [52]. However, this is rarely done in routine clinical practice.

### 7.2. Awake Endoscopy

In-office awake endoscopy with advanced HGNS programming is an efficient tool to find a more favorable airway opening, and/or an equivalent opening with increased patient comfort. Wesson et al. have proposed a protocolized approach to awake endoscopy with advanced HGNS programming [53]. The procedure requires the patient to be reclined at least 45° or to be placed in supine position. Topical anesthetic and decongestant may be used, but are not essential. Prior to the endoscopy, the functional threshold of each electrode configuration should be determined while simultaneously observing the tongue protrusion [53].

A flexible endoscope is used to evaluate upper airway opening at different settings, starting at the nasopharynx to evaluate retropalatal and oropharyngeal levels, and subsequently progressing to the oropharynx to assess retroglossal and retroepiglottic spaces. The amplitude is increased by 0.5 V for [+−+] and by 0.3 V for other electrode configurations. If discomfort is present or airway opening is insufficient, the pulse width and rate can be modified. Additionally, airway potential tests may be performed. The assessment of nasal versus oral breathing, neck position, and jaw thrust helps to guide adjunctive therapies such as the use of a chinstrap, cervical pillow or MAD therapy. When optimal settings have been found resulting in improved patient comfort and subjective benefit, a follow-up sleep study can be considered [53].

Meleca et al. performed 24 awake endoscopies in 19 patients with the most common reasons being therapy discomfort (42%), frequent therapy-related awakenings (32%), and persistent symptoms or inadequate AHI reduction (21%) [54]. Electrode configuration was changed in all patients after a first awake endoscopy. Notably, 52.6% patients had a preexisting insomnia diagnosis [54].

In a retrospective study of Wesson et al. including 17 patients, the reasons for performing an awake endoscopy were inadequate AHI reduction (88.2%) or stimulation discomfort (11.8%) [55]. An improvement in AHI was noted in 65% of patients after performing awake endoscopy with advanced programming, while 35% failed to improve. In one out of two patients with stimulation discomfort, an improvement in device usage could be achieved [55].

### 7.3. Drug-Induced Sleep Endoscopy (DISE)

DISE may serve as a valuable tool to guide further treatment decisions in case other adjustments have failed. The study of Kaffenberger et al. has proposed a decision tree for the use of DISE in patients with suboptimal HGNS therapy response and intolerance [56]. First, the effect of different HGNS settings on the opening of the upper airway can be directly visualized during DISE. Furthermore, the effect of mouth breathing can be assessed and if needed, the use of a chinstrap may be discussed with the patient [56].

The main target of HGNS therapy is the tongue base. Nevertheless, HGNS can indirectly activate other levels, such as the soft palate through a mechanism called palatoglossus coupling [62]. In the retrospective study by Kaffenberger et al., 21% of patients showed palatoglossus coupling with HGNS alone [56]. When combining HGNS with a jaw thrust maneuver, palatal coupling was present in 24% of patients. In these patients, combination therapy with MAD therapy may be considered. However, in 29% of patients no palatal coupling was present with any maneuver. In these cases, additional anterior palatoplasty may be considered. In 38% of patients with persistent oropharyngeal lateral wall collapse at multiple electrode configurations and amplitudes, expansion sphincter pharyngoplasty represents a potential solution [56].

### 7.4. Combination Therapy

Combination therapy shows promise for HGNS patients with incomplete responses, although data remains limited. A case report described the use of HGNS in combination with MAD therapy. In the reported patient, mild OSA and bothersome snoring persisted with HGNS therapy alone. When MAD therapy was added, HGNS reprogramming was necessary because of stimulation discomfort, resulting in resolution of snoring and a normalization in AHI [57]. Another case report described the combined use of HGNS, MAD, and positional therapy. In this patient, higher HGNS voltage settings were required to control respiratory events during supine REM sleep, which could not be tolerated by the patient. With the addition of MAD and positional therapy, HGNS voltage could be lowered, leading to improvements in adherence, comfort, and effectiveness [58]. A retrospective study by Patel et al. found that 39% of HGNS patients with a supine sleeping position achieved a 50% reduction in supine AHI and a post-implantation supine AHI below 15/h, suggesting that supine sleeping position may decrease HGNS treatment success [24].

In a retrospective study by Steffen et al., adjunctive uvulopalatopharyngoplasty with tonsillectomy was performed in seven HGNS non-responders that showed persistent soft palate obstruction during DISE, which resulted in an AHI reduction from 49.4 to 13.3 events/hour at 2-year follow-up [59]. Huyett et al. investigated the effect of concomitant tonsillectomy and HGNS in 19 patients with oropharyngeal lateral wall collapse, demonstrating an additional 22.9% reduction in AHI and 8.6 greater odds of achieving Sher15 criteria, compared to a control group with HGNS alone [60].

In certain patients with a suboptimal HGNS response, weight reduction might aid in improving treatment outcomes. A post hoc analysis of the ADHERE registry showed that for each 1-unit increase in BMI, there were 9% reduced odds of treatment success [61]. Furthermore, it was found that subjects with a BMI above 32 kg/m^2^ were significantly less likely to achieve Sher20 criteria [5]. A retrospective study showed that patient who gained at least 2 BMI points after HGNS had the highest postoperative AHI and the lowest AHI change compared to HGNS patients whose BMI remained stable or decreased with at least 2 BMI points [25]. Patel et al. demonstrated that patients with BMI between 32 and 35 kg/m^2^ had 75% lower odds of meeting Sher15 criteria compared with those with a BMI ≤ 32 kg/m^2^ [24].

## 8. Long-Term Management

Long-term follow-up of HGNS patients is essential to ensure long-lasting therapy success with stable therapy usage. Lenze et al. showed that HGNS device usage decreases over the first nine months after activation with an increase in the number of pauses per night [63]. In addition, Suurna et al. found that median usage per night decreased over time from 6.8 h on Day 1 to 5.8 h on Day 361, with a most pronounced decrease in patients with lowest initial usage [64]. In contrast, a recent retrospective study including 352 patients with a mean usage follow-up of 24.1 ± 17.4 months showed that time since activation had no effect on therapy usage time. Interestingly, positive predictors of HGNS usage were older age and greater number of usage days, while insomnia and higher stimulation voltage were negative predictors [65]. Data on the stimulation amplitude and how this amplitude may change over time is limited. Heiser et al. showed no significant changes in HGNS intensities over a period of 4 years, while maintaining a significant reduction in AHI [66].

## 9. Limitations of Current Evidence

The current evidence on the clinical pathway of HGNS therapy remains limited by several key methodological and clinical gaps. First of all, most available studies have a retrospective design and consist of single-center cohorts, case series, or post-market analyses with limited sample sizes. Furthermore, studies differ in type of sleep testing (HST versus polysomnography) and definitions of therapy response (titration AHI versus full-night AHI), which reduces comparability. In addition, the current evidence is limited by the inclusion of predominantly homogeneous patient populations. It is important to acknowledge these limitations, as they may affect the generalizability and overall interpretation of the results.

## 10. Conclusions

The clinical care pathway for respiration-synchronized HGNS is advancing across multiple stages, including patient selection, assessment of treatment success, and the management of incomplete responders or patients experiencing therapy-related discomfort. Each step of the clinical pathway is equally essential in ensuring therapy success. As new clinical findings emerge, the selection criteria for respiration-synchronized HGNS are changing, across anatomical markers, pathophysiological traits and comorbid sleep disorders. The criteria for defining HGNS treatment success are evolving from relying on treatment AHI obtained from in-lab titration PSG as the standard measure of HGNS efficacy to adopting a more patient-directed approach using single-amplitude, full-night sleep studies. The number of studies evaluating incomplete HGNS responders and patients experiencing therapy-related discomfort is gradually increasing, including the use of protocolized approaches for awake endoscopy and DISE with advanced HGNS programming and the role of combination therapy. However, the management of patients with insufficient treatment response remains challenging and represents an important area for future research and clinical optimization. Additional key areas for future investigation are the short- and long-term cardiovascular outcomes of HGNS therapy, the comparison of standard versus optimized clinical care pathways in multicenter clinical trials, the development of standardized PROMs to evaluate HGNS treatment efficacy, and the role of AI-driven titration and remote monitoring.

## Figures and Tables

**Table 1:** Summary of included studies regarding optimization tools for HGNS patients in the yellow pathway.

Optimization Tool for Yellow HGNS Patients	References	Study Design	Sample Size	Main Findings	Conclusion	Limitations
In-office device reprogramming	Heiser et al. [49]	Multicenter, retrospective study	14	An increased AHI reduction was found in patients with bilateral and right protrusion (Baseline: 29.6 ± 12.6/h; M06: 9.7 ± 12.6/h) compared to the mixed activation group (Baseline 49.6 ± 13.8/h; M06: 40.5 ± 4.1/h).	Postoperative tongue motions are associated with HGNS therapy outcome.	- Small sample size - Retrospective design - Short-term follow-up - Inter-rater variability in assessing tongue motion
	Steffen et al. [50]	Multicenter, prospective study	35	Changes in tongue motion patterns were frequently observed (58.8%) with different electrode configuration settings. Most of the patients alternated between right and bilateral protrusion (73.5%).	Alterations in electrode configuration modify tongue motion patterns and influence therapeutic outcomes of HGNS therapy.	- Small sample size - Inter-rater variability of tongue motion at functional thresholds and tongue motion pattern assessment
	Pawlak et al. [51]	Single-center, prospective case series	30	The upper airway opening effects were visible in all HGNS patients during endoscopy, but the effect differed for the airway level and for the applied electrode configuration.	HGNS enlarges the upper airway at a variety of electrode configurations and voltages with similar opening effects in awake endoscopy and DISE in a large number of patients.	- Small sample size - Inter-rater variability of tongue motion at functional thresholds - Inter-rater bias of endoscopy evaluation
	Kent et al. [52]	Case report	2	Office-based adjustments to respiratory sensing parameters demonstrate improvements in patient tolerance in one case and HGNS efficacy in the other case.	Respiratory sensing changes in the office setting can impact patient comfort and HGNS response.	- Small sample size (case report)
Awake endoscopy (AE) with advanced programming	Wesson et al. [53]	Protocol/single-center case series	5	Of the first 5 consecutive patients that underwent AE with advanced HGNS programming, 2 patients (40%) showed an improvement in AHI, while 3 patients (60%) failed to show improvement.	A protocolized approach to AE with advanced HGNS programming can be integrated to optimize AHI reduction and/or patient comfort.	- Small sample size (case series)
	Meleca et al. [54]	Single-center, retrospective study	60	A total of 24 AEs were performed in 19 (32%) patients. The most common complaints and reasons for AE were perceived stimulus discomfort (42%), frequent awakenings (32%), and persistent fatigue or non-normalized AHI (21%). After first AE, there was a 0.87 (53%) and 0.93 (45%) V reduction in functional threshold (FT) and minimum therapeutic amplitude (MTA), respectively.	In-office AE with HGNS advanced programming serves as a useful tool to assess the pharyngeal airway and optimize the device settings. Reduction in the FT and MTA after AE may allow for improved device compliance by reducing discomfort and frequent awakenings.	- Retrospective design - Lack of paired controls further propagates confounders
	Wesson et al. [55]	Single-center, retrospective study	17	An improvement in AHI was noted in 65% of patients after performing AE with advanced HGNS programming, while 35% failed to improve. In one out of two patients with stimulation discomfort, an improvement in device usage could be achieved.	AE with advanced HGNS programming is a powerful tool to identify settings that could increase therapy efficacy and improve patient comfort.	- Retrospective design - Small sample size
Drug-induced sleep endoscopy	Kaffenberger et al. [56]	Single-center, retrospective study	34	During DISE with HGNS, palatal coupling was observed in 7 patients (21%) with HGNS alone, 9 patients (26%) with jaw thrust alone, and 8 patients (24%) with both maneuvers combined. In 10 patients (29%), palatal coupling was absent with any maneuver. Based on DISE findings, 13 patients were recommended MAD therapy and 8 patients underwent further surgical interventions.	DISE may serve as a valuable tool to guide further treatment decisions in patients with suboptimal HGNS therapy efficacy as it identifies residual collapse.	- Retrospective design - Small sample size - Lack of sleep studies after implantation and DISE with HGNS.
Combination therapy	Lee et al. [57]	Case report	1	In a HGNS-treated patient with residual mild OSA and bothersome snoring, MAD therapy was initiated, resulting in a residual AHI of 2.1/h and resolution of snoring.	Combination of HGNS with MAD therapy may successfully treat severe OSA after incomplete response to monotherapy	- Small sample size (case report)
	Lowery et al. [58]	Case report	1	In a patient with residual REM supine OSA (AHI 17.8/h) with HGNS, the combination with MAD and positional therapy resulted in a residual AHI of 5.1/h.	Personalized combination therapy may help to optimize both AHI and subjective symptoms in patients with incomplete response to monotherapy.	- Small sample size (case report)
	Patel et al. [24]	Single-center, retrospective study	76	In adjusted analyses, HGNS patients with BMI between 32 and 35 kg/m^2^ had 75% lower odds of responding to HGNS compared with those with a BMI ≤ 32 kg/m^2^ (odds ratio, 0.25; 95% CI, 0.07–0.94). Of 44 patients who slept in a supine position, 17 (39%) achieved a treatment response, with a clinically meaningful reduction in median (IQR) supine AHI from 46.3 (33.6–63.2) events/h pre-implantation to 21.8 (4.30–42.6) events/h post-implantation.	Higher BMI and supine sleeping position may decrease therapeutic response to HGNS.	- Retrospective design - Lack of subjective rating scales - Use of HSTs for preimplantation studies - Homogeneous study population
	Steffen et al. [59]	Single-center, retrospective study	25 in total (7 patients with soft palate surgery after HGNS)	Adjunctive uvulopalatopharyngoplasty with tonsillectomy was performed in HGNS non-responders with persistent soft palate obstruction during DISE, showing an AHI reduction from 49.4 to 13.3 events/hour at 2-year follow-up.	Adjunctive soft palate surgery can improve insufficient response in HGNS patients if the obstruction is identified at the level of the velum/oropharynx.	- Small sample size - Retrospective design - Potential learning curve effect—first cases implanted at this center
	Huyett et al. [60]	Single-center, case–control study	19	Linear regression demonstrated that adding tonsillectomy resulted in an additional 22.9% [7.5, 35.2] reduction in AHI [95% confidence interval, CI] (*p* = 0.006), and 8.6 [1.7,43.4] (*p* = 0.010) greater odds [95% CI] of a successful treatment response with HGNS.	Combining tonsillectomy with HGNS may represent a promising strategy to improve success rate in patients with oropharyngeal lateral wall collapse.	- Small sample size - Potential selection bias (no randomization between tonsillectomy versus no tonsillectomy)
	Heiser et al. [61]	Multicenter, prospective observational study (ADHERE)	227	For each 1-unit increase in BMI, there was 9% reduced odds of treatment success at 1-year follow-up.	Reduced BMI is a predictor of HGNS treatment response.	- Lack of standardization in outcomes (HST versus PSG; full-night AHI versus titration AHI) - Industry-sponsored study
	Suurna et al. [5]	Multicenter, prospective observational study (ADHERE)	535 (BMI32 n = 438; BMI35 n = 97)	Surgical success was less likely in BMI35 versus BMI32 patients (59.8% vs. 72.2%, *p* = 0.02). AHI reduction in the BMI35 group was non-inferior to the BMI32 group.	Surgical response rate differs between BMI32 and BMI35 groups, while AHI and ESS reductions are similar between groups.	- Lack of standardization in outcomes (HST versus PSG; full-night AHI versus titration AHI) - Industry-sponsored study
	Han et al. [25]	Retrospective study (institutional cohort and ADHERE)	222 + 1949 patients from ADHERE	The patients who lost at least 2 BMI points had the greatest reduction in AHI (−24.77 ± 13.84, −22.58 ± 19.76 events/h) compared to patients who maintained stable BMI (−15.10 ± 20.33, −18.85 ± 18.11 events/h) or gained at least 2 + BMI points (−6.39 ± 22.52, −15.82 ± 19.63 events/h) following HGNS (*p* = 0.002 and 0.006, respectively).	Postoperative weight changes significantly correlate with HGNS response, showing the importance of adjunctive weight management after HGNS implantation.	- Retrospective design - Lack of standardization in outcomes (HST versus PSG; full-night AHI versus titration AHI) - Variability in follow-up timing - Small sample size for groups with significant BMI gain or loss

Only study results of interest to the review are reported in this table. Abbreviations: AE = awake endoscopy; AHI = apnea-hypopnea index; BMI = body mass index; DISE = drug-induced sleep endoscopy; ESS = Epworth sleepiness scale; HGNS = hypoglossal nerve stimulation; HST = home sleep test; MAD = mandibular advancement device; OSA = obstructive sleep apnea; PSG = polysomnography; REM = rapid eye movement.

## Abbreviations

The following abbreviations are used in this manuscript:

AHI	Apnea-hypopnea index
BMI	Body Mass Index
CCC	Complete concentric collapse
CPAP	Continuous Positive Airway Pressure
DISE	Drug-induced sleep endoscopy
FDA	Food and Drug Administration
HGNS	Hypoglossal nerve stimulation
HST	Home Sleep Test
IPG	Implantable Pulse Generator
MAD	Mandibular advancement device
MRI	Magnetic Resonance Imaging
OSA	Obstructive sleep apnea
PSG	Polysomnography
